# *Staphylococcus aureus* facilitates its survival in bovine macrophages by blocking autophagic flux

**DOI:** 10.1111/jcmm.15027

**Published:** 2020-01-29

**Authors:** Juan Cai, Jun Li, Yuqi Zhou, Jianqiang Wang, Jianji Li, Luying Cui, Xia Meng, Guoqiang Zhu, Heng Wang

**Affiliations:** ^1^ College of Veterinary Medicine Yangzhou University Yangzhou China; ^2^ Jiangsu Co‐innovation Center for Prevention and Control of Important Animal Infectious Diseases and Zoonoses Yangzhou China

**Keywords:** autophagy, bovine macrophage, *Staphylococcus aureus*

## Abstract

*Staphylococcus aureus* is a pathogen that is the causative agent of several human and veterinary infections and plays a critical role in the clinical and subclinical mastitis of cattle. Autophagy is a conserved pathogen defence mechanism in eukaryotes. Studies have reported that *S aureus* can subvert autophagy and survive in cells. *Staphylococcus aureus* survival in cells is an important cause of chronic persistent mastitis infection. However, it is unclear whether *S aureus* can escape autophagy in innate immune cells. In this study, initiation of autophagy due to the presence of *S aureus* was detected in bovine macrophages. We observed autophagic vacuoles increased after *S aureus* infection of bovine macrophages by transmission electron microscopy (TEM). It was also found that *S aureus*‐infected bovine macrophages increased the expression of LC3 at different times(0, 0.5, 1, 1.5, 2, 2.5, 3 and 4 hours). Data also showed the accumulation of p62 induced by *S aureus* infection. Application of autophagy regulatory agents showed that the degradation of p62 was blocked in *S aureus* induced bovine macrophages. In addition, we also found that the accumulation of autophagosomes promotes *S aureus* to survive in macrophage cells. In conclusion, this study indicates that autophagy occurs in *S aureus*‐infected bovine macrophages but is blocked at a later stage of autophagy. The accumulation of autophagosomes facilitates the survival of *S aureus* in bovine macrophages. These findings provide new insights into the interaction of *S aureus* with autophagy in bovine macrophages.

## 1 INTRODUCTION

Mastitis results in low milk production and poor milk quality, causing significant economic loss to the dairy industry.^1^, ^2^ Bovine mastitis is divided into clinical and subclinical mastitis, and the incidence of subclinical mastitis is much higher than that of clinical mastitis.^3^, ^4^ Although mastitis is caused by several pathogens, *Staphylococcus aureus* intramammary infection (IMI) is the main cause of subclinical mastitis. *Staphylococcus aureus*, originally thought to be an extracellular pathogen, has been shown to invade various types of professional and non‐professional phagocytic cells and therefore may be a facultative intracellular pathogen.^5^, ^6^, ^7^, ^8^, ^9^

Autophagy, a fundamental cellular homeostatic mechanism, is an intracellular degradation/recycling system in eukaryotic cells and transports damaged cytoplasm and organelles to lysosomes for degradation. Effective autophagy is dependent on the balance between autophagosome formation and elimination, and any defects in the autophagy pathway can cause autophagy dysfunction. In addition, there is substantial evidence showing that autophagy dysregulation can lead to mammalian diseases.^10^, ^11^, ^12^

The innate immune system is essential for *S aureus* clearance.^13^ Professional phagocytic cells, including macrophages, are the first line of defence against pathogens. In bovine mastitis, macrophages present in the mammary glands and the acinar cells, protect the epithelium from invading pathogens.^14^ Recent studies have shown that *S aureus* can survive in different types of macrophages. *Staphylococcus aureus* were detected in neutrophils isolated from mouse bone marrow leucocytes by Gresham HD et al.^15^ In addition, Elliott et al have demonstrated short‐term survival of *S aureus* in human alveolar macrophages.^16^ Studies have shown that the ability of *S aureus* to survive being phagocytosed by human macrophages may contribute to the spread of infection and may be harmful to the host.^17^ Hebert A et al have shown the presence of viable *S aureus* in macrophages in milk samples from animal with bovine mastitis.^18^ Intracellular survival of *S aureus* may be responsible for the chronic persistence of infection in bovine subclinical mastitis and contribute to the spread of *S aureus* to other cows and herds.

Previous researches have proved that autophagy is involved in the intracellular survival of *S aureus*, but autophagosome escape and intracellular survival of *S aureus* in different cells has been controversial. For example, Schnaith et al reported that *S aureus* uses autophagosomes as a replicating niche in HeLa cells.^19^ Mestre et al 20 confirmed that *S aureus* escaped from autophagosomes to the cytoplasm for replication. Previously, we have shown that the formation of autophagosomes facilitates the replication of *S aureus* in bovine mammary epithelial cells.^21^ The survival strategies of the pathogen are as diverse as strains or host cell types used.^7^, ^22^ Although reports on *S aureus* and autophagy are increasing, none proves whether *S aureus* escapes or subverts autophagy in bovine macrophages. Insufficient understanding of the interaction of *S aureus* with autophagy in different host cells limits the development of new therapeutic strategies for *S aureus* induced mastitis.

In this study, we aimed to reveal whether the autophagic flux is unobstructed in *S aureus‐*infected bovine macrophages, and the relationship between the patency of autophagic flux and the intracellular survival of *S aureus*. We demonstrate for the first time that *S aureus* can block autophagic flux and promote its survival in bovine macrophages. This study provides new insights into the interaction of *S aureus* with autophagy in bovine macrophages and provides new insights into the prevention and treatment of *S aureus* infection.

## 2 MATERIALS AND METHODS

### 2.1 Bovine macrophage cell culture

Bovine macrophage cells were cultured in RPMI 1640 medium (Gibco) supplemented with 10% heat‐inactivated foetal bovine serum (Gibco), at 37°C with 5% CO_2_.

### 2.2 Preparation of *S aureus*

*Staphylococcus aureus* (ATCC29213) was cultured overnight at 37°C in 20 mL liquid Luria‐Bertani (LB) (Tryptone 10 g/L, Yeast extract 5 g/L, and NaCl 10 g/L). Upon reaching the logarithmic growth phase, the bacteria were washed with phosphate‐buffered saline (PBS) thrice and diluted with RPMI 1640 medium to achieve bacterial concentration for multiplicity of infection (MOI = 1:1).

### 2.3 Usage of autophagy regulatory agents

3‐Methyladenine (3‐MA) (Sigma‐Aldrich) was stored as a 250 mM stock solution in PBS, Rapamycin (Rap) (Sigma‐Aldrich) was stored as a 5 mM stock solution in DMSO, and Chloroquine (CQ) (Sigma‐Aldrich) was stored as a 10 mM stock solution in PBS. One hour before infection, cells were pre‐treated with 3‐MA (2.5 mM), Rap (2.5 μM), and CQ (40 μM). Thereafter, the cells were infected with *S aureus* for 2 hours. Subsequently, *S aureus* was removed, and fresh medium was added. At the indicated time, cells were collected and the relevant index was tested.

### 2.4 Western blot analysis

The cells were seeded in 6‐well plates with 1 × 10^6^ cells/well. When the cells were confluent, they were administered appropriate treatment, harvested and lysed in RIPA buffer supplemented with protein phosphatase inhibitors (Applygen Technologies Inc) and protease inhibitors (Applygen Technologies Inc). Total protein was separated by SDS‐PAGE and transferred to a PVDF membrane (Millipore). Subsequently, the LC3 membrane was blocked overnight at 4℃ in TBST containing 10% skim milk. The β‐actin and p62 membranes were blocked for 1 hours at 25℃ in TBST containing 5% skim milk. The membrane was then hybridized with specific antibodies, including anti‐LC3 (MBL), and anti‐p62 and anti‐β‐actin (Cell Signaling Technology Inc). The LC3 membrane was then incubated with HRP‐conjugated goat anti‐mouse IgG (MBL), and β‐actin and p62 membranes were incubated with HRP‐conjugated goat anti‐rabbit IgG (Cell Signaling Technology Inc). All blots were detected with enhanced chemiluminescence (ECL; Vazyme).

### 2.5 Immunofluorescence staining

Cells were seeded on glass coverslips for 12‐14 hours. When the cells had grown to 80% confluence, the cells were treated according to the experimental requirements, fixed with 4% paraformaldehyde, and permeabilized with Triton X‐100. Therefore, the cells were incubated with anti‐LC3 antibody for 1 hours at room temperature, washed three times with phosphate‐buffered saline (PBS) for 5 miutes each and incubated with the FITC‐conjugated secondary antibody for another 1 hours. Finally, the cells were stained with DAPI. After washing thrice with PBS, LC3 protein puncta were examined by Leica SP8 laser scanning confocal microscopy (Leica TCS SP8 STED, Leica Corp.).

### 2.6 Transmission electron microscopy

The cells were fixed with 2.5% glutaraldehyde at 4℃ overnight, fixed in 1% citric acid for 1 hours, dehydrated in a gradient of ethanol and embedded in resin. Ultrathin sections (100 nm) were prepared and stained with uranyl acetate and lead citrate. Thereafter, the sections were observed with a transmission electron microscope (FEI Tecnai Spirit Bio TWIN).

### 2.7 Plasmid transfection

Cells were seeded on glass coverslips for 12 hours. When the cells had grown to 80% confluence, 1 μg/mL GFP‐RFP‐LC3 plasmid was used to transiently transfect the cells using Lipofectamine 3000, according to the manufacturer's instructions. After 2 hours, the cells were incubated in RPMI 1640 medium containing 15% FBS for 24 hours. Thereafter, the bovine macrophages were infected with *S aureus* for 2 hours. Subsequently, the cells were collected, fixed with 4% paraformaldehyde, and the nuclei were labelled with DAPI. Finally, fixation was performed using a fluorescent fixative, and co‐localization was evaluated by Leica SP8 laser scanning confocal microscopy ( Leica TCS SP8 STED, Leica Corp.).

### 2.8 Determination of bacterial intracellular survival

Bovine macrophages were co‐cultured with *S aureus* for 2 hours. Thereafter, the cells were cultured for 1 hours in RPMI 1640 medium supplemented with 100 μg/mL gentamicin sulphate to kill extracellular bacteria. Afterwards, the culture medium was discarded, and cells were washed thrice with PBS. Subsequently, the cells were cultured in RPMI 1640 medium for 0, 1, 2 and 3 hours separately and lysed with 0.5% Triton X‐100 (in PBS) for 10 minutes. Series dilution of the lysate was inoculated onto LB agar plates, and colonies were counted to determine colony forming units (cfu) of the intracellular bacteria.

### 2.9 Statistical analysis

Statistical analysis was performed using SPSS 17.0 using one‐way ANOVA and Dunnett's test. Data represent mean ± SD from triplicate independent experiments. Values of *P* < .05 between two sets of data were considered to be statistically significant.

## 3 RESULTS

### 3.1 Autophagy was triggered by *S aureus* in bovine macrophages

**Figure 1:**
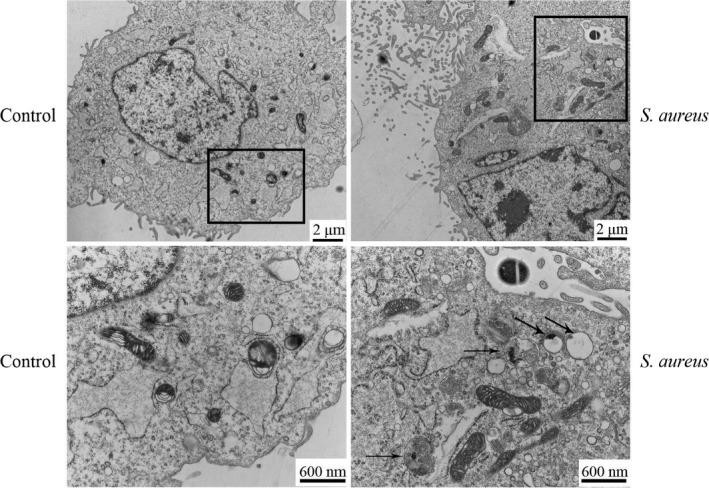
Autophagy in bovine macrophage cells was triggered by *Staphylococcus aureus*. Bovine macrophages were infected with *S aureus* for 2 h (MOI = 1:1). Samples were prepared as described in Materials and Methods and examined under transmission electron microscopy (TEM). Scale bar = 2 μm or 600 nm

*Staphylococcus aureus* can cause autophagy in the infected cells.^23^ Autophagosomes, with the bilayer membrane structure, and autolysosomes, with the monolayer membrane structure, were observed by transmission electron microscopy, as the gold standard for detecting autophagy.^24^ To detect whether autophagy occurred after *S aureus* infection, we infected bovine macrophages with *S aureus* (MOI = 1:1) for 2 hours. It can be seen that the number of autophagic vacuoles increased significantly upon infection (Figure 1). Ultrastructural analysis showed that autophagy occurred after *S aureus*‐infected bovine macrophages.

### 3.2 Expression of LC3 proteins in bovine macrophage cells induced by *S aureus* was enhanced

**Figure 2:**
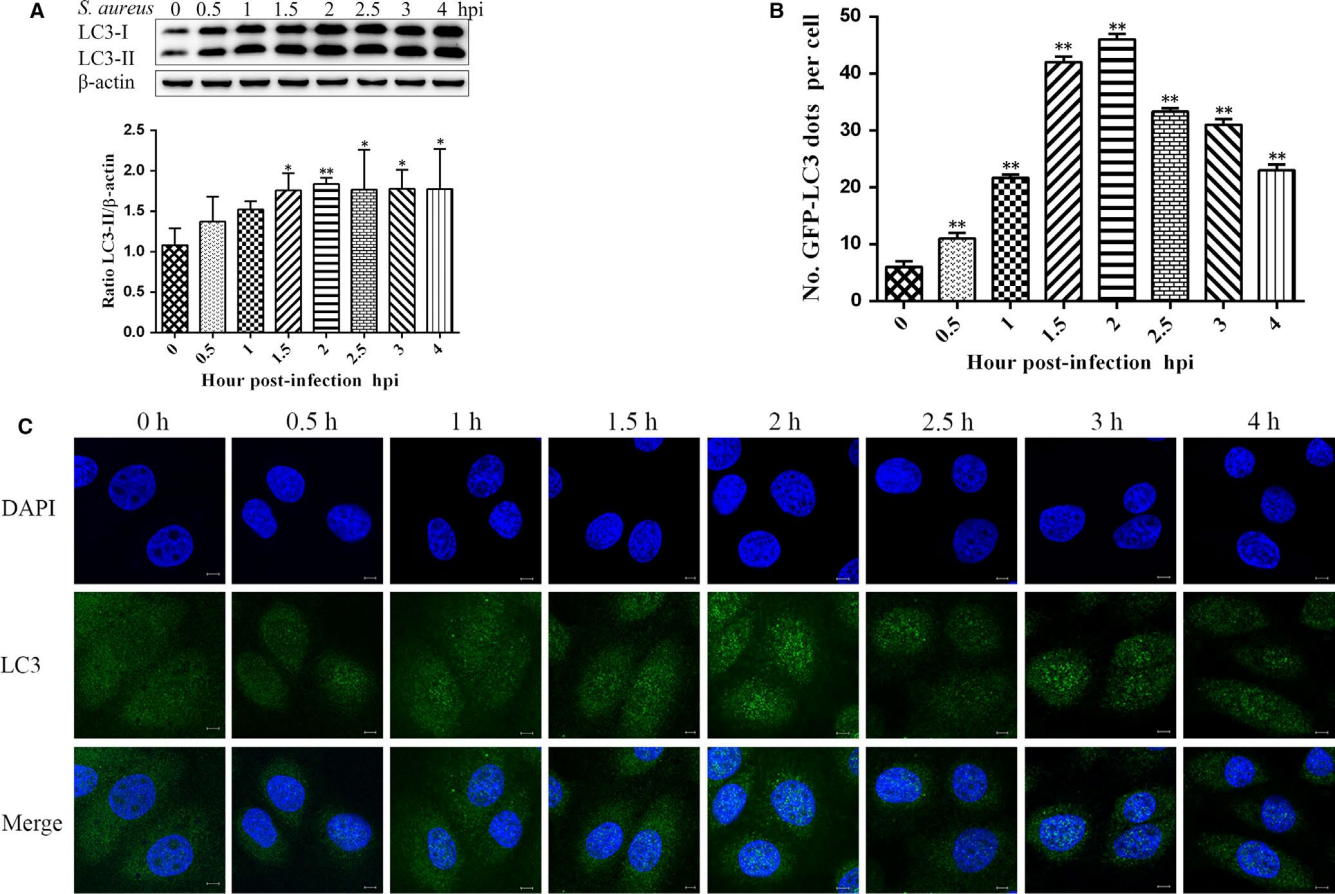
Enhanced expression of LC3 in *Staphylococcus aureus*‐stimulated bovine macrophage cells. A, Bovine macrophages were infected with *S aureus* (MOI = 1:1). Cells were harvested at the indicated times and subjected to Western blot analysis using antibodies. After *S aureus* infects bovine macrophages, the expression of LC3‐II protein is increased. B, The average number of LC3 puncta per cell is displayed. C, Immunofluorescence assay was used to further observe changes in LC3 expression in *S aureus*‐infected bovine macrophages. Bovine macrophages were grown on coverslips in 24‐well plates and infected at a MOI = 1:1. Cells were fixed at different time points after infection and incubated with anti‐LC3 antibody to visualize LC3 particles. Scale bar = 10 μm. The results are representative of three independent experiments. Statistical significance was determined by Student's *t* test (*P < .05; **P < .01)

Studies have shown that autophagy can cause an increase in LC3 expression.^24^ To investigate whether autophagy occurs after *S aureus* infects bovine macrophages, we used Western blot to assess the expression of LC3‐II at different time points (0, 0.5, 1, 1.5, 2, 2.5, 3, 4 hours) after infection. We found that *S aureus* significantly induced LC3‐II protein expression at 1.5, 2.5, 3 and 4 hours compared with 0 hours. The increase in the expression level of LC3‐II protein induced at 2 hours was extremely highly significant (Figure 2A). To further investigate the changes in LC3 after *S aureus* infection of bovine macrophages, we used direct fluorescence detection to detect the number of LC3 puncta. Compared with the 0 hours time point, the number of LC3 puncta significantly increased after *S aureus*‐infected bovine macrophages (Figure 2B,C).

### 3.3 Degradation of autophagy substrate p62 and autophagosome‐lysosomal fusion were arrested after *S aureus* infection of bovine macrophages

**Figure 3:**
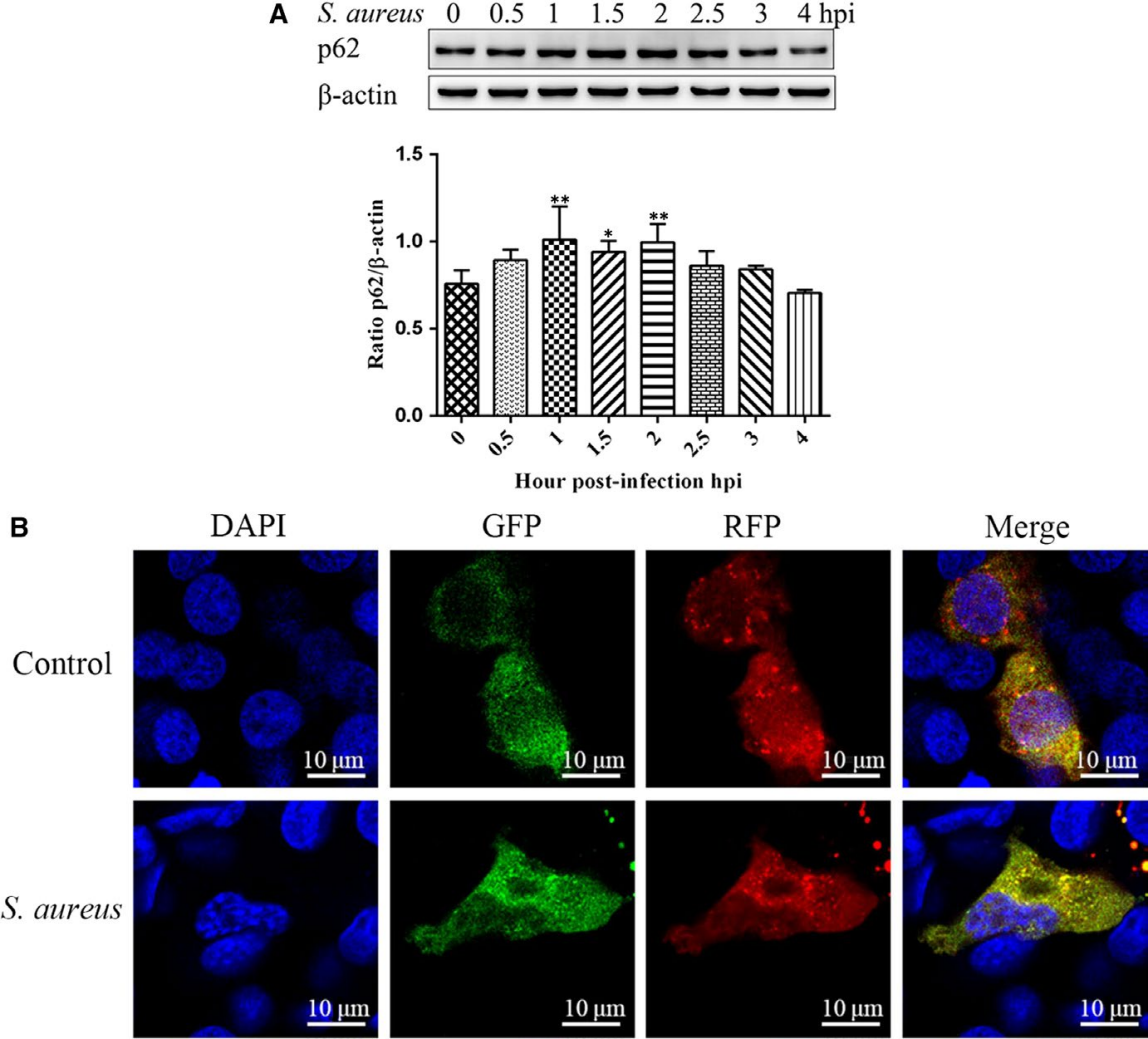
Degradation of autophagy substrate p62 and autophagosome‐lysosomal fusion were arrested after *Staphylococcus aureus* infection of bovine macrophages. A, Bovine macrophages were infected with *S aureus* (MOI = 1:1) at the indicated time points. The expression level of p62 was measured by immunoblotting. Relative p62 expression level was calculated, after being normalized to β‐actin, using ImageJ. B, Cells grown on glass coverslips were transfected with GFP‐RFP‐LC3 plasmid for 24 h and then treated with *S aureus* MOI = 1:1 for 2 h. Autophagy flow was evaluated by confocal microscopy. The results are representative of three independent experiments. Statistical significance was determined by Student's *t* test (*P < .05; **P < .01)

Elevated expression of LC3‐II is associated with autophagy activation and the level of LC3‐II is an indicator of the amount of autophagosomes formed. After *S aureus* infects bovine macrophages, the expression of LC3‐II is increased. Given that the autophagosome is an intermediate structure in a dynamic pathway, the number of autophagosomes observed at any specific time point is a function of the balance between the rate of their generation and the rate of their conversion into autolysosomes.^24^ Therefore, an increase in the number of autophagosomes may be either due autophagic induction or an inhibition of pathways of autophagy. p62 has an LC3 interaction region and acts as an autophagy receptor for degradation of ubiquitinated substrates.^25^ To test the changes of autophagic flux in macrophages infected with or without *S aureus*, expression levels of the autophagy‐specific substrate p62 (SQSTM1) were detected at the different time by Western blot. The expression of p62 was significantly increased at 1.5 hours after infection with bovine macrophages by *S aureus*, and the increase in p62 expression was particularly significant at 1 and 2 hours (Figure 3A). The results showed that the degradation of p62 was blocked after *S aureus*‐infected bovine macrophages. Autophagosomes fuse with lysosomes to form autolysosomes for the degradation of internalized cargo.^26^ Autophagosome‐lysosome fusion is an essential step in autophagic flux. To further understand the effect of *S aureus* infection on autophagic flux in macrophages, dynamic analysis of autophagy flux assessed by GFP‐RFP‐LC3 plasmid morphologically was used. Using this tandem construct, LC3‐II positive autophagosomes are simultaneously labelled with GFP and RFP signals marked with yellow spots. After fusion with lysosomes, autolysosomes are shown as red spots only, because GFP loss of fluorescence at acidic pH.^26^ As we can see, the yellow spots increased and the red spots decreased in the *S aureus* treated group compared to the blank group (Figure 3B). This indicates that *S aureus* infection of bovine macrophages leads to a flow block in autophagic flux.

### 3.4 Effects of autophagy regulatory agents on different stages of autophagy in *S aureus*‐infected bovine macrophages

**Figure 4:**
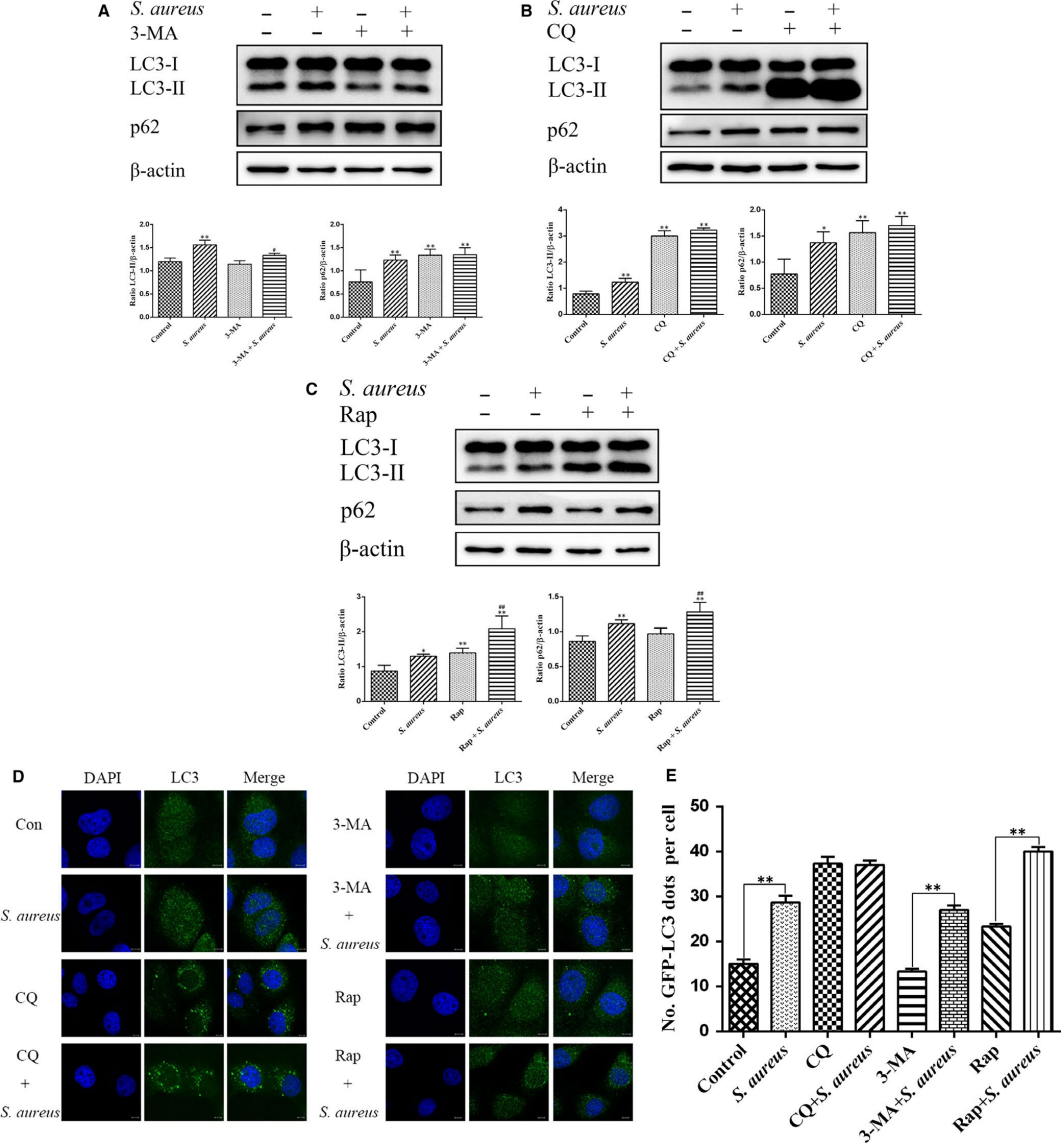
Degradation of p62 was blocked after *Staphylococcus aureus* infection in bovine macrophage cells. A, Effect of early‐autophagy inhibitor (3‐MA) on LC3 and p62. Bovine macrophages were infected with *S aureus* (MOI = 1:1) for 2 h in the presence or absence of 3‐MA (2.5 mM). Cells were harvested and subjected to Western blot analysis. Relative protein expression levels of LC3 and p62 were calculated after being normalized to β‐actin using ImageJ. B, Effect of late‐autophagy inhibitor (CQ) on LC3 and p62. Bovine macrophages were infected with *S aureus* (MOI = 1:1) for 2 h in the presence or absence of CQ (40 μM). Cells were harvested and subjected to Western blot analysis. Relative protein expression levels of LC3 and p62 were calculated after being normalized to β‐actin using ImageJ. C, Effect of autophagy promoter (Rap) on LC3 and p62. Bovine macrophages were infected with *S aureus* (MOI = 1:1) for 2 h in the presence or absence of Rap (2.5 μM). Cells were harvested and subjected to Western blot analysis. Relative protein expression level of LC3 and p62 was calculated after being normalized to β‐actin using ImageJ. D, Immunofluorescence analysis of intracellular LC3 protein levels. Bovine macrophages were infected with *S aureus* (MOI = 1:1) in the presence or absence of an autophagy regulatory regulator for 2 h and stained with mouse anti‐LC3 monoclonal antibody and FITC‐labelled fluorescent secondary antibody. E, The average number of LC3 puncta per cell is displayed. Scale bar = 10 μm. The results are representative of three independent experiments. Statistical significance was determined by Student's *t* test. Compared with the control group (*P < .05; **P < .01), compared with the modulator of the autophagy group (#*P* < .05; ##*P* < .01)

Autophagy regulatory agents are widely used in the study of various diseases.^27^, ^28^ To further evaluate the effect of *S aureus* infection on autophagy in bovine macrophages, we used autophagy regulators at different stages of autophagy (early‐autophagy inhibitor, 3‐MA, late‐autophagy inhibitor, CQ, and autophagy inducer, Rap). Western blot results showed that the 3‐MA and *S aureus* co‐treatment group lead to an increase in the LC3 expression and showed no change in p62 expression compared with the 3‐MA‐treated group (Figure 4A). The CQ and *S aureus* co‐treatment group did not show change in the expression levels of LC3 and p62 compared with the CQ‐treated group (Figure 4B). Furthermore, the Rap and *S aureus* co‐treatment group showed significantly increased expression of LC3 and p62 compared with the Rap‐treated group (Figure 4C). To further confirm the reliability of Western blot results, we used direct immunofluorescence to detect changes in LC3 puncta of *S aureus*‐infected bovine macrophages, using autophagy regulators. Immunofluorescence results were consistent with Western blot results (Figure 4D,4). Compared to the 3‐MA‐treated group, the 3‐MA and *S aureus* co‐treatment group showed increased expression of LC3, and the CQ and *S aureus* co‐treatment group did not show altered expression levels of LC3 compared with the CQ‐treated group. Furthermore, Rap and the *S aureus* co‐treatment group showed a significant increase in the expression of LC3 compared with the Rap‐treated group. All these results suggest that *S aureus* both induces autophagy and blocks autophagy flux, leading to the accumulation of autophagosomes, in bovine macrophages.

### 3.5 Blocking autophagic flux facilitates the survival of *S aureus* within cells

**Figure 5:**
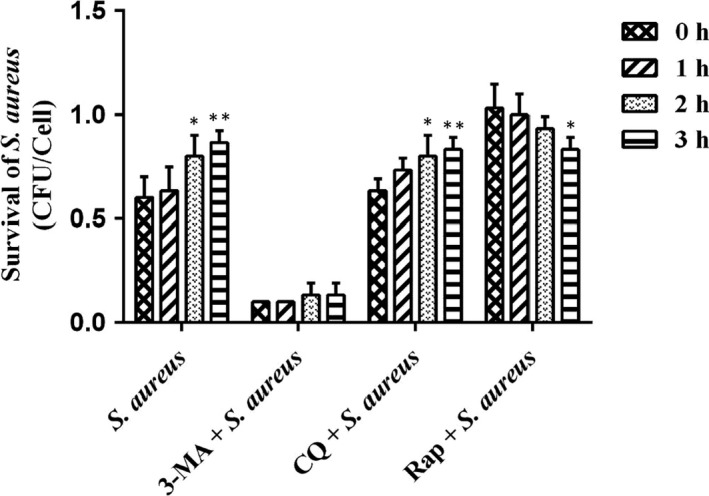
Blocking autophagic flux facilitates the survival of *Staphylococcus aureus* within cells*.* After pretreatment of bovine macrophages with autophagy regulatory agents (2.5 mM 3‐MA, 40 μM CQ, 2.5 μM Rap) for 1 h, bovine macrophages were infected with *S aureus* (MOI = 1:1) for 2 h. The cells were cultured with 100 μg/mL gentamicin sulphate for 1 h to kill the extracellular bacteria. Cell lysates were collected at 0, 1, 2 and 3 h, and serial dilutions were plated on LB solid medium to determine the amount of CFU/mL. The data represent three different experiments. Data are expressed as mean ± SD from three independent experiments. Compared to the 0 h (*P < .05, **P < .01)

Results from our studies show that when *S aureus*‐infected bovine macrophages for 2 hours, accumulation of autophagosomes increased significantly. The literature reports that *S aureus* can invade and replicate in many types of phagocytic and non‐phagocytic cells.^23^ However, it is not clear whether the accumulation of autophagosomes contributes to the survival of intracellular bacteria. In order to study whether the accumulation of autophagosomes caused by *S aureus*‐infected bovine macrophages is conducive to its survival in cells, we pre‐treated cells with different stages of autophagy regulators. After the bovine macrophages were infected with *S aureus* for 2 hours, the extracellular bacteria were killed with gentamicin sulphate, and then the number of intracellular bacteria was detected at different time points (0, 1, 2, 3 hours). Figure 5 shows that in the *S aureus* infection group, the number of intracellular bacteria increased significantly at 2 and 3 hours of treatment as compared with 0 hours. There was no change in the number of intracellular bacteria in 1, 2 and 3 hours compared with 0 hours in the autophagy pre‐inhibitor 3MA treatment group. In the CQ treatment group of post‐autophagy inhibitors, compared with 0 hours, the number of intracellular bacteria increased significantly at 2 hours, and the number of intracellular bacteria increased significantly at 3 hours. In the autophagy promoter Rap treatment group, the number of intracellular bacteria decreased significantly at 3 hours compared with 0 hours. The results show that the accumulation of autophagosomes increases the number of viable intracellular bacteria.

## 4 DISCUSSION

Chronic subclinical mastitis induced by *S aureus* has caused huge economic losses to the global dairy industry, and antibiotic‐resistant bacteria that threaten global public health have also been reported.^29^ However, there are no effective controls for *S aureus* mastitis so far and further research is needed. Autophagy is a cellular homeostatic mechanism in eukaryotes, which is as an effective mechanism for survival during cellular stress and also as an anti‐infective mechanism.^30^ In recent years, autophagy has been extensively studied as a defence mechanism against pathogen. There have been many reports on autophagy induction by pathogens in humans and other animal models.^23^, ^31^ However, there is little information about autophagy in bovine immune cells during bacterial infection. Although there have been many reports that *S aureus* can survive in immune cells of different species,^16^, ^18^ there is little information on autophagy of bovine immune cells during pathogen infection. In addition, the interaction between pathogens and different host cells is widely controversial.^7^, ^32^ Investigation on the relationship between *S aureus* and autophagy in bovine immune cells is of great significance for the prevention and treatment of mastitis in dairy cows. In this study, we demonstrated the effect of *S aureus* on autophagy in bovine macrophages during infection, for the first time.

Microtubule‐associated light chain protein 3 (LC3) is an autophagosome‐specific membrane marker in mammalian cells.^33^ When autophagy occurs, LC3‐I, which is expressed on the cytoplasm and is coupled to phosphatidylethanolamine to form LC3‐II, localizes to the inner and outer membranes of the autophagosome. Therefore, the levels of LC3‐II expression, detected by Western blotting, can indicate the level of autophagy.^24^ In the present study, the expression level of LC3‐II began to increase at 1.5 hours during *S aureus* infection and was the highest at 2 hours. Moreover, immunofluorescence staining showed that the fluorescence intensity of the LC3 puncta varied with infection time and was the strongest at 2 hours. These results are similar to the results observed in RAW264.7 cells.^31^ Thus, *S aureus* induces autophagy in bovine macrophages. Further, p62 is a substrate for autophagy and is selectively incorporated into autophagosomes by direct binding to LC3, which is efficiently degraded by autophagy.^34^ Evaluating autophagic flux involves two aspects: whether autophagosomes gradually increase after autophagic activation or whether autophagosomes fuse to and degrade within the lysosomes, which would result in a decrease in p62. Thus, the expression level of p62 is inversely proportional to autophagic flux. In this study, protein expression of p62 increased in bovine macrophages at 1, 1.5 and 2 hours, induced by *S aureus*. Moreover, yellow spots increased and red spots decreased after *S aureus* infection. The above results indicated that *S aureus‐*infected bovine macrophages caused autophagic flow block.

To further clarify the effect of *S aureus* on autophagy of bovine macrophages, we used modulators to regulate the changes in autophagic flux.^24^ Western blot analysis showed that the expression of LC3 increased with *S aureus* infection after 3‐MA treatment but there was no significant change in p62, compared with the 3‐MA group. Compared with CQ treatment, there was no significant change of LC3 and p62 levels upon *S aureus* infection after CQ treatment. Infection with *S aureus* after Rap treatment further enhanced the expression of LC3 and p62 compared with the Rap group, which was also verified by direct immunofluorescence detection. Thus, infection induced by *S aureus* blocked late autophagy and inhibited the degradation of autophagosomes, which was similar to the action of the late‐autophagy blocker, CQ and indicated that the increase in autophagosomes after *S aureus* infection in bovine macrophages is due to the blockade of autophagic flux.

Notably, autophagy is bidirectional during bacterial infection. In infected cells, autophagosome‐enclosed bacteria were delivered to the lysosomes and degraded, which can inhibit bacterial replication and transmission to protect organisms from further damage. However, some bacteria have evolved an autophagic escape mechanism and can survive in cells for a long time. They can even utilize the autophagosomes for replication. *Shigella flexneri* has been shown to recruits Toca‐1 via IcsB to escape autophagy recognition.^35^ PrfA regulatory factors of Listeria monocytogenes, including ActA and phospholipases PI‐PLC and PC‐PLC, allow cytosolic bacteria to escape lysosomal degradation.^36^ In this study, *S aureus* blocked autophagic flux and inhibited autophagosome degradation in bovine macrophages. Other researches have also verified that *S aureus* could utilize autophagy to survive in cells.^37^, ^38^ Survival of *S aureus* in immune cells may be an important mechanism of persistent infection in cow mastitis. Previous studies have shown that inhibition of autophagy with wortmannin decreased intracellular proliferation of *S aureus in Hela cells*, while Rap induced autophagy doubles the number of intracellular bacteria.^19^ However, we found that the number of intracellular *S aureus* is gradually increased with CQ treatment, while *S aureus* replication in the cells is gradually reduced when Rap is used to enhance autophagy and autophagic flux is not blocked. Recently, it has been reported that in the *S aureus*‐infected murine fibroblasts NIH/3T3, *S aureus* escapes phagocytic vacuoles and prevents further degradation by MAPK14/p38a MAP kinase‐mediated autophagy blockade.^30^ This difference may be due to the different escape mechanisms of *S aureus* in different cells.

Overall, *S aureus* infection activated autophagy in bovine macrophages, but blocked the accumulation of autophagosomes in the late stage of autophagy. Moreover, the accumulation of autophagosomes is conducive to the survival of *S aureus* in the cells. This provides insights into the interaction mechanism between *S aureus* and bovine immune cells to develop new treatments for *S aureus* induced mastitis.

## 5 CONCLUSION

Autophagy is widely regarded as a host defence mechanism that contributes to the degradation of intracellular pathogens.^39^ However, many intracellular pathogens have developed elaborate mechanisms to disrupt or even utilize autophagic structures to survive in mammalian host cells.^40^ *S aureus* can evade macrophages by autophagic flux to survive in bovine macrophages. However, for the prevention and treatment of chronic *S aureus* induced mastitis further research is needed to elucidate the detailed molecular mechanism of how *S aureus* escape bovine macrophages.

## CONFLICT OF INTEREST

The author(s) declare no potential conflicts of interests with respect to the research, authorship and/or publication of this article.

## AUTHOR CONTRIBUTIONS

Heng Wang conceived and designed the project and revised the manuscript. Juan Cai and Jun Li participated in experimental design, planned the experiments, performed the experiments, integrated the data and wrote the manuscript. Yuqi Zhou and Jianqiang Wang performed the experiments. Jianji Li, Luying Cui, Xia Meng and Guoqiang Zhu provided technical support.

## Data Availability

I confirm that my article contains a Data Availability Statement even if no data is available (list of sample statements) unless my article type does not require one (e.g., Editorials, Corrections, Book Reviews, etc.). I confirm that I have included a citation for available data in my references section, unless my article type is exempt.
